# On the Use of Multivariate Methods for Analysis of Data from Biological Networks

**DOI:** 10.3390/pr5030036

**Published:** 2017-07-03

**Authors:** Troy Vargason, Daniel P. Howsmon, Deborah L. McGuinness, Juergen Hahn

**Affiliations:** 1 Department of Biomedical Engineering, Rensselaer Polytechnic Institute, Troy, NY 12180, USA; 2 Center for Biotechnology and Interdisciplinary Studies, Rensselaer Polytechnic Institute, Troy, NY 12180, USA; 3 Department of Chemical and Biological Engineering, Rensselaer Polytechnic Institute, Troy, NY 12180, USA; 4 Department of Computer Science, Rensselaer Polytechnic Institute, Troy, NY 12180, USA; 5 Department of Cognitive Science, Rensselaer Polytechnic Institute, Troy, NY 12180, USA

**Keywords:** multivariate statistics, Fisher discriminant analysis, probability density function, autism spectrum disorder, one carbon metabolism, transsulfuration, urine toxic metals, classification, machine learning

## Abstract

Data analysis used for biomedical research, particularly analysis involving metabolic or signaling pathways, is often based upon univariate statistical analysis. One common approach is to compute means and standard deviations individually for each variable or to determine where each variable falls between upper and lower bounds. Additionally, *p*-values are often computed to determine if there are differences between data taken from two groups. However, these approaches ignore that the collected data are often correlated in some form, which may be due to these measurements describing quantities that are connected by biological networks. Multivariate analysis approaches are more appropriate in these scenarios, as they can detect differences in datasets that the traditional univariate approaches may miss. This work presents three case studies that involve data from clinical studies of autism spectrum disorder that illustrate the need for and demonstrate the potential impact of multivariate analysis.

## 1. Introduction

Statistical analysis is a critical component for supporting any finding—whether from a clinical trial or other data collection. While there are numerous types of scenarios where such an analysis may need to be applied, two expository examples are: (1) when a clinical trial tests measurements from two or more populations, such as healthy versus diseased or placebo versus treatment; or (2) when a patient’s blood sample is analyzed and the measured values are compared against reference ranges for a healthy individual. In both cases, the analysis is typically performed by comparing the representative value of one specific measured quantity against the same measured quantity of others, and this comparison is typically done for each measured quantity. However, such an approach will ignore correlations that may exist between the different measured quantities. If the measured quantities are representative of activity in a biological network where components are connected via reactions, interactions, or regulatory effects, such as in metabolic or signaling pathways, then traditional univariate approaches will potentially misrepresent the true behavior of the system under investigation. Multivariate analysis can address this shortcoming and, more accurately, it can be used to elucidate the characteristics of a biological network.

The value of considering multiple quantities simultaneously is recognized in the medical and biomedical communities, as demonstrated by the use of measurement ratios for univariate statistical analysis. The ratio of S-adenosylmethionine (SAM) to S-adenosylhomocysteine (SAH), for example, is used as an indicator of DNA methylation capacity [1]. Kidney functioning can be assessed with the ratio of blood urea nitrogen to creatinine in the plasma [2]. Furthermore, the ratio of total cholesterol to high-density lipoprotein cholesterol is used to provide an assessment of cardiovascular health [3]. Using ratios or observing the statistical distribution of ratios, instead of analyzing the separate values individually, can be advantageous as the interactions between different biological components may then be considered. However, the ability to take correlations of a larger number of measurements into account, without needing to specify the relationships, would be of even greater benefit. Multivariate statistical methods, such as Fisher Discriminant Analysis (FDA) [4] and its nonlinear extension, Kernel Fisher Discriminant Analysis (KFDA) [5], are promising options as they can address the aforementioned drawbacks of univariate analytical approaches.

This paper provides three case studies that compare the results obtained from univariate and multivariate statistical analyses of data from clinical studies. These case studies illustrate the benefits of using multivariate techniques over their univariate counterparts. While one must be careful when drawing conclusions from specific case studies about a more general setting, this work is nevertheless intended to highlight examples of advantages that can be gained by using multivariate analysis techniques, especially in cases where biological networks are involved.

## 2. Preliminary Information

### 2.1. Univariate Statistical Analysis

Univariate analyses are those aiming to summarize the characteristics of a single variable. These produce the statistics commonly reported in scientific literature, including the mean, standard deviation, and quantiles. When comparing a single measurement between two study populations, such as placebo and treatment groups, the two-sample *t*-test can be used to test for a significant difference in the group means, provided that the measurement is normally distributed in both groups [6]. Alternatively, the Mann-Whitney *U* test allows one to test for a significant difference in medians between two identical, but shifted, distributions [7].

### 2.2. Multivariate Statistical Analysis

Multivariate analysis involves the investigation of multiple variables simultaneously and encompasses a number of techniques that can be used to model data arising from complex systems. Such techniques take on a variety of forms and are used for a number of different tasks. For example, analyses of variance models [8] are commonly used to test the effects of multiple categorical factors on a measured response variable. The support vector machine [9] is a popular option for the supervised classification of groups of data consisting of a number of measurements. Additionally, hierarchical clustering [10] can be used for cluster analysis, while partial least squares regression [11] offers an approach for parameter estimation. Multivariate methods are often implemented in machine learning tasks in which models are developed with existing data and then used to predict new data. FDA is a useful method for maximizing separation between two or more groups of data samples [4] and is most appropriate when the input variables are continuous and normally distributed [12].

**Figure 1:**
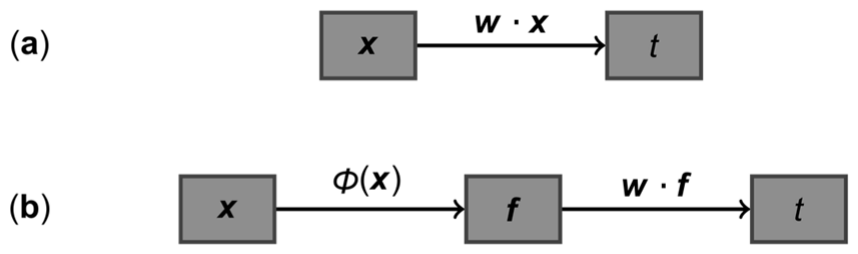
Schematics of the transformations used in Fisher Discriminant Analysis (FDA) and Kernel Fisher Discriminant Analysis (KFDA): (**a**) In FDA, the dot product of vector ***w*** with data sample ***x*** is calculated to obtain the projected value *t*; (**b**) KFDA first maps each sample ***x*** to a higher-dimensional space ***f*** according to the nonlinear transformation *φ*(***x***). The dot product of ***w*** with ***f*** (rather than with ***x***) is then calculated to obtain the projection *t*.

The input of FDA is a set of data samples ***X***, where each sample ***x*** is a vector containing a fixed number of measurements. With a two-class problem (again consider the placebo versus treatment example), a subset of these samples ***X*_1_** belongs to one class while the remaining subset of samples ***X*_2_** belongs to the other class. The purpose of FDA is to calculate the projection vector ***w***, which transforms each ***x*** to a single score variable *t*, that best separates the samples in ***X*_1_** and ***X*_2_**. Separability is quantified by *J*, the ratio of the between-class scatter to the within-class scatter, and ***w*** is chosen to maximize this quantity [4]. Figure 1a summarizes this linear transformation performed in FDA as applied to individual samples.

The principle of KFDA is similar to that of FDA, except that KFDA is capable of modeling nonlinear relationships between input variables rather than just linear ones. Before calculating a projection direction ***w*** to best separate ***X*_1_** and ***X*_2_**, KFDA first applies a nonlinear transformation to each ***x***, expressed as ***f*** = *φ*(***x***), to map each to a higher-dimensional variable space ***f***. Since the explicit mapping of *φ*(***x***) is not known, an implicit mapping can be defined such that the inner product between any two *φ*(***x***) is a Mercer kernel [5]. In a two-class problem, all ***f*** belonging to one class make up ***F*_1_** while the ***f*** in the other class comprise ***F*_2_**. The vector ***w*** that best separates ***F*_1_** and ***F*_2_** is then determined, with the linear projection *t* = ***w***·***f*** capturing nonlinear relationships in the original variable space of ***x***. Like FDA, nonlinear KFDA also aims to maximize the value of *J*. A schematic of the operations involved in KFDA is provided in Figure 1b. It should also be noted that the radial basis function, a commonly-used kernel, will be used in this work.

## 3. Advantages of Multivariate Approaches for Biological Network Analysis

**Figure 2:**
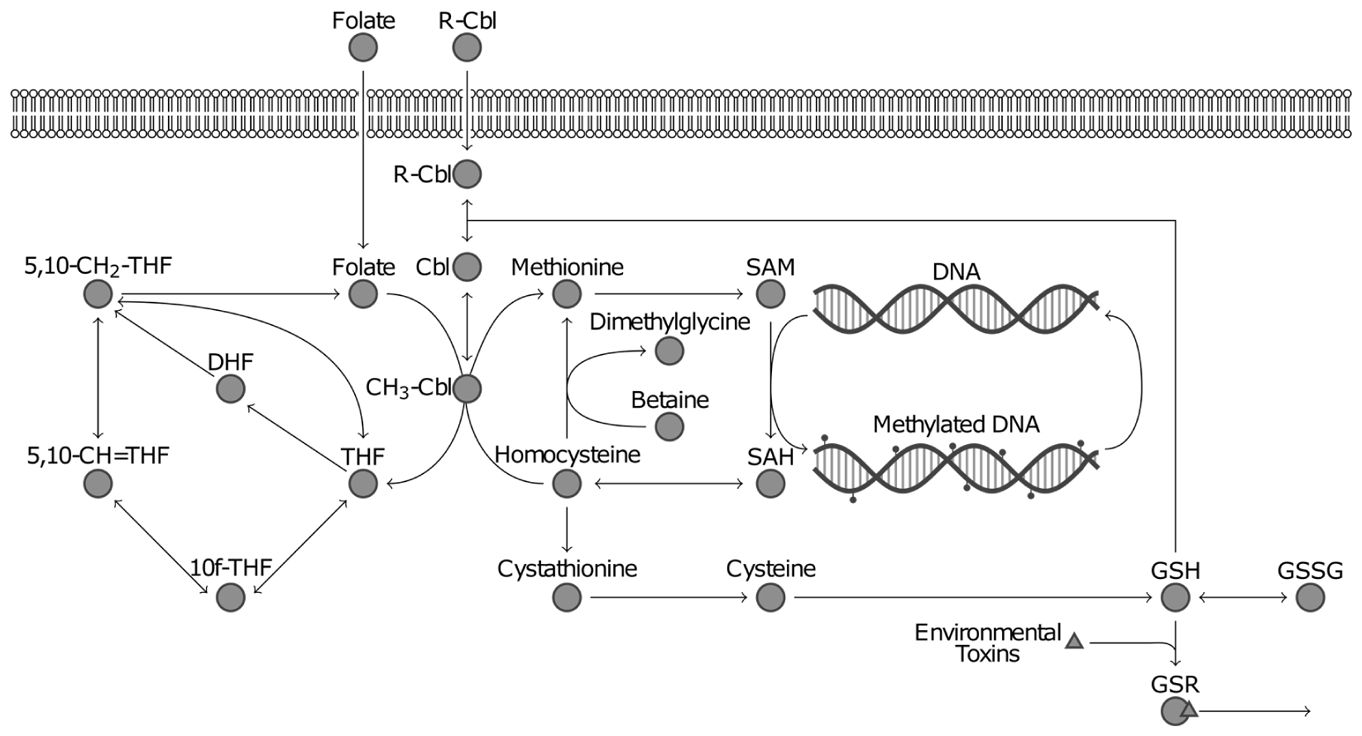
Diagram of major metabolites and reactions involved in the folate-dependent one-carbon metabolism (FOCM) and transsulfuration (TS) pathways. DNA methylation plays an important role in epigenetics and glutathione (GSH) is responsible for the clearance of environmental toxins.

Three case studies are presented in this section that illustrate some benefits of using multivariate approaches to analyze biological networks. The focus of these case studies is on folate-dependent one-carbon metabolism (FOCM) and transsulfuration (TS), two metabolic pathways with critical roles in the human body (Figure 2). FOCM, which occurs in every cell type [13], is involved with the epigenetic control of gene expression through DNA methylation [14]. The TS pathway, initiated by the conversion of homocysteine to cystathionine, is found in the liver, kidney, pancreas, small intestine, and brain, and contributes to the management of intracellular oxidative stress [15,16]. The FOCM and TS pathways are connected and together form an important juncture in the larger metabolic networks of human cells.

FOCM and TS are believed to be closely intertwined with genetic and environmental factors associated with autism spectrum disorder (ASD) predisposition [17] and therefore are often the focus of clinical studies investigating metabolic abnormalities in ASD [18–20]. These studies have found the ratio of S-adenosylmethionine (SAM) to S-adenosylhomocysteine (SAH) [21] to be reduced in individuals with ASD compared to neurotypical (NT) peers, which suggests a reduced DNA methylation capacity. The same studies have determined an increased proportion of oxidized to reduced glutathione [22], an important antioxidant, to indicate an irregular balance between oxidants and antioxidants (redox status) in ASD.

The case studies that follow will highlight three unique aspects of multivariate analysis. First, the utility of incorporating multiple measurements for assessing network activity will be demonstrated using a general example. Second, advantages of using multivariate over univariate methods to analyze FOCM/TS metabolite data will be studied in the context of ASD classification. Third, the ability of nonlinear multivariate approaches to uncover relationships that linear analyses cannot describe will be explored, with a focus on measurements of toxic metals from the urine of individuals with ASD.

### 3.1. Advantages of Using Multiple Correlated Measurements for Diagnosis: A General Case

**Figure 3:**
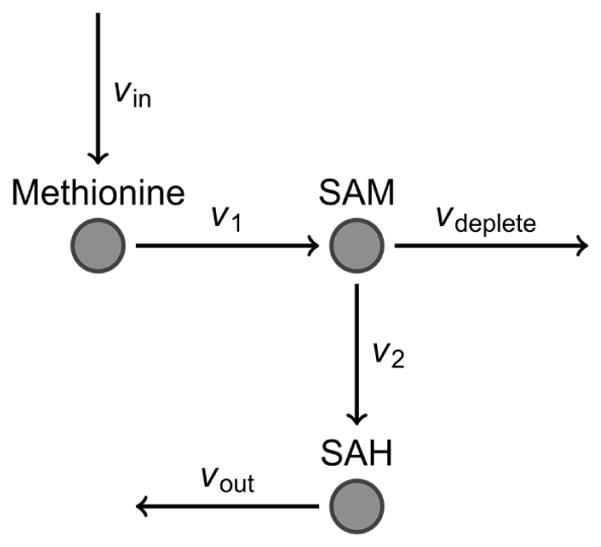
A simplified representation of a subset of reactions in FOCM responsible for DNA methylation.

Consider a subset of reactions in FOCM associated with DNA methylation to be represented by the model in Figure 3. This model is taken to describe FOCM activity in liver cells. The metabolic reactions are assumed to proceed according to mass action kinetics and the reaction rates are thus proportional to the concentrations of the substrates, similar to the FOCM/TS model design used in a previous study [23]. In this model, methionine is delivered to liver cells at the rate *v*_in_. Methionine is converted to SAM at a rate *v*_1_ by methionine adenosyltransferase enzymes. SAM is then converted to SAH by methyltransferase enzymes at the rate *v*_2_, or is depleted by other reactions and excreted at a rate described by *v*_deplete_. Finally, SAH is converted to other FOCM products at a rate *v*_out_.

Recall that a reduced SAM/SAH ratio has been observed in individuals with ASD and indicates a lowered capacity for DNA methylation. In the context of the metabolic model, this implies one of five scenarios: (1) reduced SAM and relatively normal SAH; (2) relatively normal SAM and elevated SAH; (3) both reduced SAM and elevated SAH; (4) elevated SAM and further elevated SAH; or (5) reduced SAH and further reduced SAM. In each scenario, the measurement of both SAM and SAH is required to make an informed assessment about DNA methylation capacity. Therefore, measuring SAM or SAH alone will not provide sufficient information to form meaningful conclusions about methylation status.

For example, suppose a patient has significantly increased *v*_in_, which can be due to a number of reasons. All modeled metabolite concentrations (methionine, SAM, SAH) will then increase with time, along with their associated reaction rates. Clinical measurement of SAH sometime afterwards will indicate an elevated concentration of SAH, and following scenario (2) or scenario (3) the unwary clinician might conclude that the patient has a decreased SAM/SAH ratio. However, with an additional measurement of SAM it would be discovered that the SAM concentration is also elevated and the SAM/SAH ratio is relatively unchanged. The only way to verify this is to incorporate multiple measurements into the diagnosis and obtain a bigger picture of the network being studied.

A potential alternative to this multivariate approach would be to develop a comprehensive network model of the metabolic pathways under investigation and analyze the behavior of the network as a whole. While this can provide correlational (and sometimes causal) information that a multivariate statistical approach cannot, it also has several drawbacks. For one, a network model requires reasonably extensive knowledge of the network’s structure and properties, which are not always known, or a very large dataset to construct the network’s structure. Understanding the network’s behavior then necessitates that the measurements be available for a large number of components of the network, whereas a multivariate analysis can be performed with just a subset of these measurements and without specifying the relationships between individual components. The presented multivariate approach thus offers a simplified, yet effective, representation of the network that can serve as a biomarker for the disorder or disease of interest.

### 3.2. Advantages of Using Multivariate Approaches over Univariate Approaches: Application to ASD Classification Using Clinical Measurements of FOCM/TS Metabolites

The purpose of this case study is to illustrate the benefit of incorporating multiple measurements, rather than a collection of individual ones, into a procedure for classifying two groups of data. To demonstrate this point, data from the Integrated Metabolic and Genomic Endeavor (IMAGE) study at Arkansas Children’s Hospital Research Institute [20] will be used. The IMAGE study investigates plasma profiles of FOCM and TS metabolites in individuals with ASD and how they compare to those of NT individuals. Measurements of primary interest in this study are methionine cycle and TS metabolites, as well as DNA methylation and oxidative stress markers.

ASD classification has been performed with high accuracy by applying FDA to measurements from the IMAGE study [24]. In a multivariate analysis of these data, a subset of five measurements was found to provide excellent classification of the ASD and NT cohorts. These measurements, which are explained elsewhere in greater detail [20], were: (1) the percentage of DNA that is methylated (% DNA methylation), an indicator of epigenetic activity; (2) the concentration of 8-hydroxyguanosine, a marker of oxidative damage in DNA; (3) the concentration of glutamylcysteine, the precursor for glutathione; (4) the ratio of free oxidized cysteine to free reduced cysteine (free cystine/free cysteine), an indicator of extracellular redox status; and (5) the percentage of glutathione molecules that are oxidized (% oxidized glutathione).

**Figure 4:**
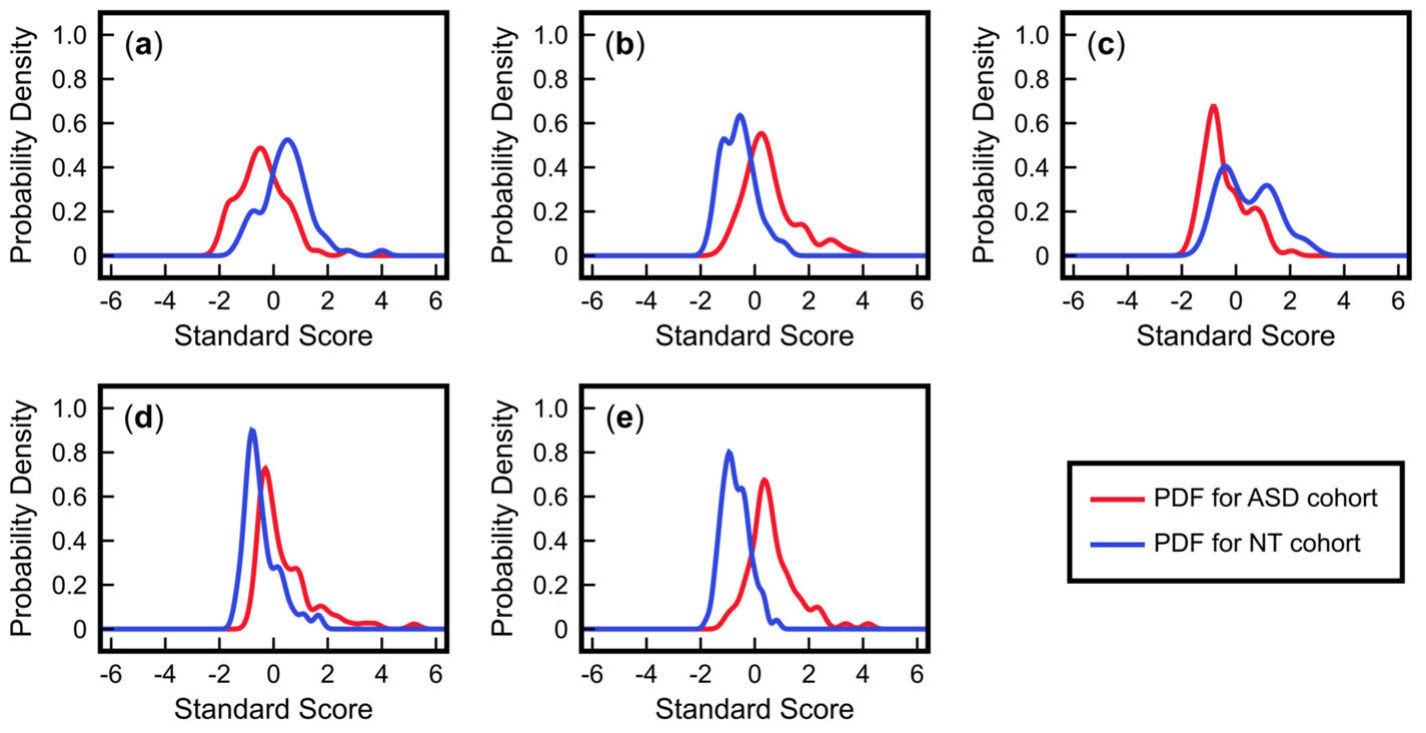
Probability density functions (PDFs) of five measurements for ASD and NT cohorts from the IMAGE study: (**a**) % DNA methylation; (**b**) 8-hydroxyguanosine; (**c**) glutamylcysteine; (**d**) free cystine/free cysteine; (**e**) % oxidized glutathione. These PDFs are based on the standardized values of each measurement (i.e., all samples for a measurement are scaled such that the mean value is 0 and the standard deviation is 1).

Table 1 provides descriptive statistics for each of these measurements in the ASD and NT cohorts, along with *p*-values from the two-tailed Welch’s *t*-test (significance level *α* = 0.05). These numbers indicate a significant difference in the mean between the cohorts for all five measurements. To further characterize these differences, the probability density functions (PDFs) of each variable were plotted for each group (Figure 4). The differences in means between cohorts are apparent in these distributions. However, there is still significant overlap of the PDFs, suggesting that these measurements will not allow for an accurate classification of a patient when considered individually.

**Figure 5:**
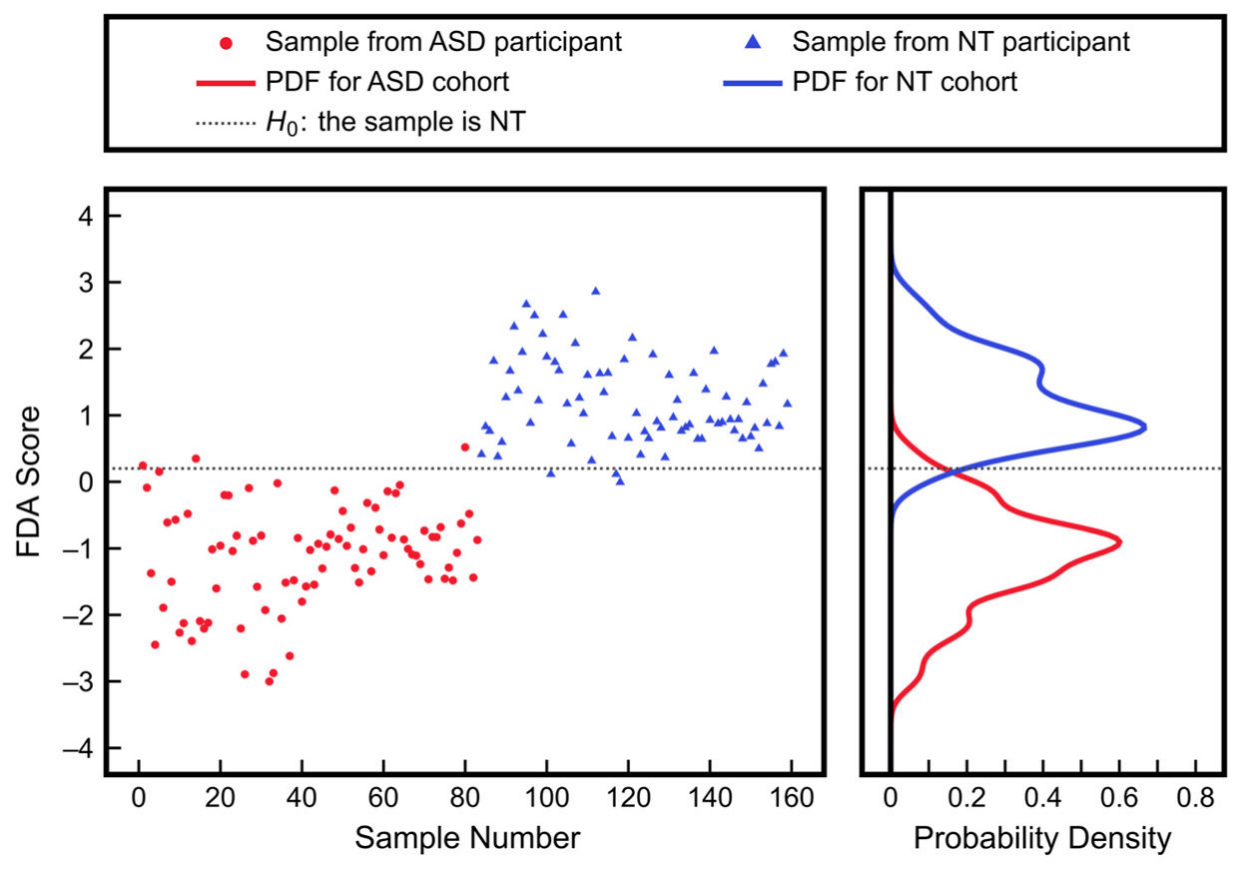
Multivariate analysis with FDA using five measurements from the IMAGE study (% DNA methylation, 8-hydroxyguanosine, glutamylcysteine, free cystine/free cysteine, and % oxidized glutathione). The scores are the projected values obtained by leave-one-out cross-validation with FDA, while the PDFs were obtained by fitting to the scores. The shown threshold corresponds to a Type I error of 4.8% and a Type II error of 5%.

The use of multivariate methods such as FDA can address this issue. Figure 5 shows the results of applying FDA to these five measurements using leave-one-out cross-validation [25]; this method provides an independent assessment of the model performance by training the FDA model on all samples but one, obtaining a projected score for the left-out sample, and then repeating this process such that every sample has been left out exactly once. The resulting PDFs for the ASD and NT cohorts are well-separated, and when the indicated threshold is used for classification, the corresponding Type I and Type II errors are only 4.8% and 5%, respectively. It must be emphasized that since cross-validation was used in this analysis, the problem of potentially overfitting the FDA model by including more variables was addressed; these results also indicate the model’s ability to accurately predict new data points that were not originally used to develop the model.

In summary, univariate analysis of the five FOCM/TS measurements indicates significant differences in the means between the ASD and NT cohorts for each of the measurements. However, due to the variance in the measurements, these differences are not sufficiently large for purposes of classification. On the other hand, the application of a multivariate technique (in this case, FDA) allows us to simultaneously consider all of these measurements and determine a pattern in the data that can accurately predict if measurements come from a participant in the ASD or NT cohort.

### 3.3. Advantages of Nonlinear Approaches over Linear Approaches: Application to ASD Classification Using Clinical Measurements of Urine Toxic Metals

This final case study examines how nonlinear multivariate methods can uncover relationships among measurements that linear methods are unable to capture. The advantages of these nonlinear approaches have previously been shown using measurements of urine toxic metals that were collected as part of the Comprehensive Nutritional and Dietary Intervention Study at Arizona State University [26]. These data are again considered here.

Recall that the TS pathway is responsible for the synthesis of glutathione, which plays a major role in the regulation of oxidative stress. One use of glutathione is to aid with the removal of unwanted substances, such as toxic metals, from the body by binding them and subsequently facilitating excretion. Most of the excretion is done via feces [27], although other routes such as excretion via urine can also play a role [28]. Given that children with ASD have been found to have reduced levels of glutathione [18], it is likely that their toxic metal excretions will be different from those of their neurotypical peers. Thus, urine toxic metals can potentially be used as an indicator of FOCM and TS abnormalities in patients with ASD.

Descriptive univariate statistics for measurements of three urine toxic metals collected in the Comprehensive Nutritional and Dietary Intervention Study [26] are given in Table 2. It should be noted that each measurement is normalized by the amount of creatinine to address the varying dilution of each urine sample. Among these urine toxic metals, none had means that were significantly different between the ASD and NT cohorts when evaluated with the two-tailed Welch’s *t*-test (significance level *α* = 0.05). This univariate analysis suggests little to no separability between the ASD and NT groups based on these three measurements.

**Figure 6:**
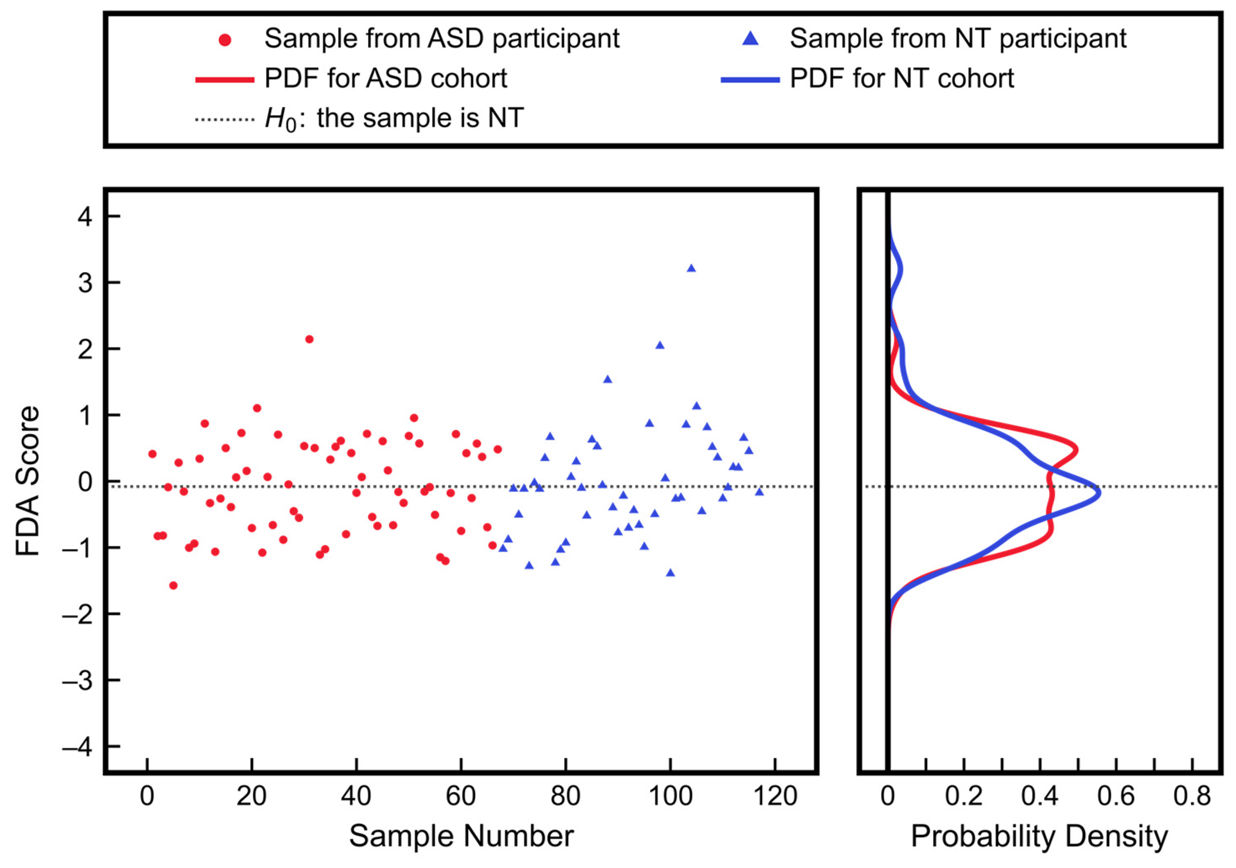
Results of classification using linear FDA with three urine toxic metal measurements (aluminum, cesium, tungsten) as inputs. FDA scores were from leave-one-out cross-validation and the PDFs were obtained by fitting to the scores. The Type I and Type II errors are both 50%.

Applying FDA to these data does not produce any substantial separation between cohorts either (Figure 6). The PDFs resulting from leave-one-out cross-validation overlap almost entirely, with the corresponding Type I error at 50% and Type II error also at 50%. Using a linear multivariate approach thus does not offer any additional insights for classification. This is not unexpected, as the results of the univariate analysis also showed minimal differences between the ASD and NT measurements. However, there may be nonlinear relationships present that neither univariate nor linear multivariate techniques can describe.

**Figure 7:**
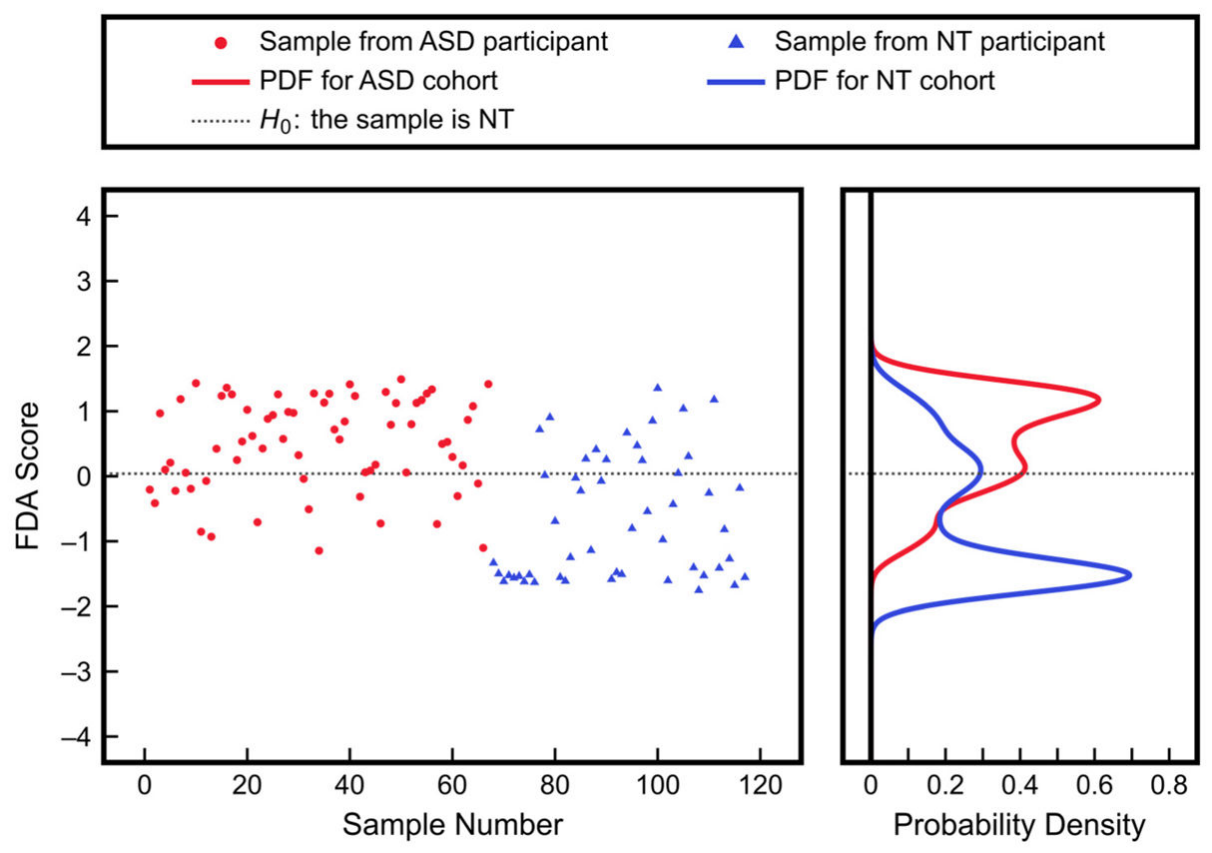
Results of classification using nonlinear KFDA with three urine toxic metal measurements (aluminum, cesium, tungsten) as inputs. KFDA scores were from leave-one-out cross-validation and the PDFs were obtained by fitting to the scores. The corresponding Type I and Type II errors are 29% and 28%, respectively.

Using nonlinear KFDA with these three urine toxic metal measurements improves the classification significantly, as seen in Figure 7. The PDFs after leave-one-out cross-validation with KFDA produce Type I and Type II errors of 29% and 28%, respectively. These results are notably better than those obtained from the linear analysis, though still far from being usable as a diagnostic tool. This inability to accurately classify the two cohorts highlights that KFDA will not detect strong differences between groups of data that are very similar, as is the case with the three urine toxic metal measurements presented here. It is nevertheless important to note that the nonlinear approach was still able to identify certain differences in the patterns in the data between groups that the linear analysis missed. This example highlights that a univariate or a linear approach being unable to find differences between two groups does not mean that differences may not exist. This is especially so for more complex relationships between variables that may be present in biological networks.

## 4. Conclusions

Statistical analysis is an integral part of any clinical trial and is also critical for evaluating medical laboratory test results. While the current state of practice in many areas of biomedical research involving metabolic or signaling pathways is to use univariate statistical analysis to evaluate one measurement at a time (across a cohort where this is applicable), this approach is sub-optimal when the measured quantities are correlated in some form, as is the case when they are connected via a biological network. This work included three case studies involving clinical data to demonstrate that significant advantages can be gained from using multivariate statistical analysis on these types of data. It is the opinion of the authors that multivariate analysis techniques should be more broadly considered for measurements taken from biological networks.

## Figures and Tables

**Table 1:** Means and standard deviations of five FOCM/TS measurements for the autism spectrum disorder (ASD) and neurotypical (NT) cohorts from the Integrated Metabolic and Genomic Endeavor (IMAGE) study. Reported *p*-values were obtained from the two-tailed Welch’s *t*-test.

Measurement	ASD Mean ± SD *n* = 83	NT Mean ± SD *n* = 76	*p*-Value
% DNA methylation	3.37 ± 0.87	4.26 ± 0.90	<0.001
8-hydroxyguanosine (pmol/mg DNA)	89.2 ± 27.9	56.7 ± 17.9	<0.001
glutamylcysteine (μM)	1.87 ± 0.46	2.37 ± 0.59	<0.001
free cystine/free cysteine	1.51 ± 0.58	1.06 ± 0.35	<0.001
% oxidized glutathione	0.22 ± 0.07	0.12 ± 0.04	<0.001

**Table 2:** Means and standard deviations of levels of three urine toxic metals in the ASD and NT cohorts from the Comprehensive Nutritional and Dietary Intervention Study. Metal levels are in units of μg/g of creatinine. Reported *p*-values were obtained from the two-tailed Welch’s *t*-test.

Measurement	ASD Mean ± SD *n* = 67	NT Mean ± SD *n* = 50	*p*-Value
Aluminum	9.03 ± 6.55	8.55 ± 11.15	n.s.
Cesium	4.03 ± 1.92	3.74 ± 1.75	n.s.
Tungsten	0.29 ± 0.25	0.29 ± 0.21	n.s.
